# Dr. Edmondston, on Ophthalmia

**Published:** 1807-04

**Authors:** Arthur Edmondston


### To the Editors of the Medical and Phyſical Journal.

GENTLEMEN,

I Have just read Dr. Vetch's "Account of the Ophthalmia, which has appeared in England since the return of the British army from Egypt." The facts which he records, confirm the idea of the contagious nature of that disease, yet I am sorry to observe, he appears to have misunderstood the nature and extent of my opinions on that subject. With the decisions of criticism I shall never contend, but in silence receive its censure or its praise; but, however ungracious the task is, I conceive it necessary to correct mistake, and anticipate, if possible, future misconception.

The first instance, where my name occurs in his book, is in a note on page 27, where the author having refuted the whimsical notions of some travellers, respecting the production of ophthalmia, states in one general observation, that I consider the disease to be uniformly communicated by one individual looking at the disordered eyes of another; and he refers the reader to a pamphlet which I published four years before on this subject. I confess, I was a little surprised at seeing the pamphlet noticed at all, since in a larger work, which appeared early in September last year, I had investigated the point at considerable length, and had endeavoured to appreciate the force and influence of the very instances alluded to.

In page 122, this larger work is mentioned; adverting to the opinion just stated, and anxious to shew that we essentially differ in our view of the propagation of ophthalmia, Dr. Vetch observes, "the support which it has received from the inquiry prefixed to Mr. Edmondston's Treatise on the varieties and consequences of ophthalmia, justly entitled it to consideration. This author, who stated the same opinion less fully, in the treatise I have already noticed, admits the infectious nature of the discharge from the eyes; at the same time, by a number of observations on the abstract nature of contagion, he endeavours to explain its propagation upon the principle of some more subtile effluvia, arising either from the system, or the eye of the patient." This very passage acknowledges the latitude which I have given to my opinion, and destroys the belief in that unity of explanation, which Dr. Vetch conceives I maintain.

In

In the pamphlet on Ophthalmia, which I published in 1802, I detailed faithfully, the facts which had presented themselves to my observation, whether they appeared to favour or to oppose my opinion. But even then, I referred the disease which occurred in the Argyleshire Regiment, chiefly to the operation of *fomites* retained by the bedding which the Guards had occupied on their voyage from Egypt to Gibraltar, and which bedding was used by the Argyleshire on its return from Gibraltar to England.

In the Inquiry, prefixed to my Treatise on the varieties and consequences of Ophthalmia, I repeated every fact as it formerly stood in the pamphlet, because I conceived such conduct would impress the reader with a belief in my adherence to truth, which I considered its chief merit. I added however, such facts as had occurred to myself in the interval, or which I had acquired from others; and to assume no more than what I might with justice lay claim to, I prefaced my investigation with an historical sketch of the opinions of those who had preceded me; and by quoting the different passages connected with the subject, endeavoured fairly to appreciate the relative value and importance of each. Although I considered my account of the Egyptian ophthalmia as its most prominent feature, and that variety of the disease which I had had the most frequent opportunities of observing; yet it was my wish to divest the work of that locality which is necessarily attached to the consideration of any individual variety of a disease, and I hoped that my views of its nature and history would apply to the occurrence of ophthalmia in every age and country.

In attempting, therefore, to deduce from the facts the general principle that ophthalmia is propagated by contagion or infection, I began by making observations on the origin and habitudes of action of different contagions, and by analogical arguments, founded on comparative views of epidemic and contagious diseases, endeavoured to shew that there was no positive evidence to invalidate the belief, that any disease, however local, might under certain circumstances acquire the power of propagating itself. This I thought might remove some of the difficulties which had hitherto opposed the general adoption of the opinion. But in attempting to ascertain the laws which regulate the propagation of ophthalmia, I never restricted my explanation to any mode of operation, because I was aware, before certainty could be attained on that head, some peculiar laws remained to be discovered.

Hence,

Hence, although from the history of the ophthalmia, which appeared at Paris in 1803, and from some other facts stated by authors, I was led to suppose that it might be propagated, in some instances by the emission from the body of a peculiar matter, communicated through the medium of the atmosphere; yet not satisfied with this explanation as applied to every case, I have expressed my conviction, that in regiments, the scene of Dr. Vetch's experience, it is propagated by the direct application of matter from the eyes. In the second part of my Inquiry, pages 45 and 6, the following observations occur. "The influence of this contagion, (that of ophthalmia) does not appear to extend far from the source of its evolution, as for a considerable time after the first appearance of the disease, it seemed to require almost immediate contact to communicate it to others; for it was confined to the soldiers, two of whom always sleeping in one bed, the sick and the healthy are often indiscriminately mingled together. It is probable therefore, from this circumstance, that a great proportion of cases of this malignant ophthalmia occurring in regiments are occasioned by the direct application of virus to the eyes. Those individuals who have attentively guarded against too near an approach, have in general completely escaped."—And again, "As the discharge of morbific matter, however, from the eyes, in cases of virulent ophthalmia, is continual and abundant, an ample source of infection is constantly present; and without extreme care and attention to avoid intercourse with the bedding or cloths of those labouring under it, the disease may be unconsciously propagated to a wide extent."

These passages, I conceive, state explicitly, my conviction of the very limited range of the ophthalmic infection. Indeed it has been, and it is my opinion, that in almost every instance it requires contact to produce the effect. But there are facts which are not easily reconciled with this explanation; and therefore, in a philosophical inquiry into a subject, certainly novel, and where the opinions stated were in a great measure peculiar, I thought it would be presuming too far to hazard my unqualified belief in any particular mode of operation. Satisfied with having established on incontrovertible evidence, the principle, that ophthalmia is propagated by contagion or infection, I left the demonstration of particular laws to time, and the observation of others. I never therefore can consider the better developement of any individual point comprehended in my proposition, as disclosing a doctrine different from my own, but as tending more fully to confirm the general truth of it.

I am, &c.

ARTHUR EDMONDSTON.

*February* 21, 1807.

